# Capsaicin: A Potent Inhibitor of Carbonic Anhydrase Isoenzymes

**DOI:** 10.3390/molecules190710103

**Published:** 2014-07-10

**Authors:** Betul Arabaci, Ilhami Gulcin, Saleh Alwasel

**Affiliations:** 1 Atatürk University, Faculty of Sciences, Department of Chemistry, Erzurum 25240, Turkey; E-Mail: btlozer@hotmail.com.tr; 2 Zoology Department, College of Science, King Saud University, Riyadh 11451, Saudi Arabia; E-Mail: salwasel@ksu.edu.sa

**Keywords:** capsaicin, carbonic anhydrase, enzyme purification, enzyme inhibition, affinity chromatography

## Abstract

Carbonic anhydrase (CA, EC 4.2.1.1) is a zinc containing metalloenzyme that catalyzes the rapid and reversible conversion of carbon dioxide (CO_2_) and water (H_2_O) into a proton (H^+^) and bicarbonate (HCO_3_^–^) ion. On the other hand, capsaicin is the main component in hot chili peppers and is used extensively used in spices, food additives and drugs; it is responsible for their spicy flavor and pungent taste. There are sixteen known CA isoforms in humans. Human CA isoenzymes I, and II (hCA I and hCA II) are ubiquitous cytosolic isoforms. In this study, the inhibition properties of capsaicin against the slow cytosolic isoform hCA I, and the ubiquitous and dominant rapid cytosolic isozymes hCA II were studied. Both CA isozymes were inhibited by capsaicin in the micromolar range. This naturally bioactive compound has a Ki of 696.15 µM against hCA I, and of 208.37 µM against hCA II.

## 1. Introduction

Carbonic anhydrase (CA) enzymes are virtually ubiquitous in all living systems and have important roles in pH regulation, carboxylation reactions, fluid balance, bone resorption, tumorigenicity, calcification, the synthesis of bicarbonate and in many other pathological and physiological processes [1,2,3,4]. CA catalyzes the reversible hydration of carbon dioxide (CO_2_) and water (H_2_O) to bicarbonate (HCO_3_^−^) and a proton (H^+^) [5,6,7,8,9].

CO_2_ + H_2_O ⇔ H_2_CO_3_ ⇔ HCO_3_^−^ + H^+^

**Scheme 1:**
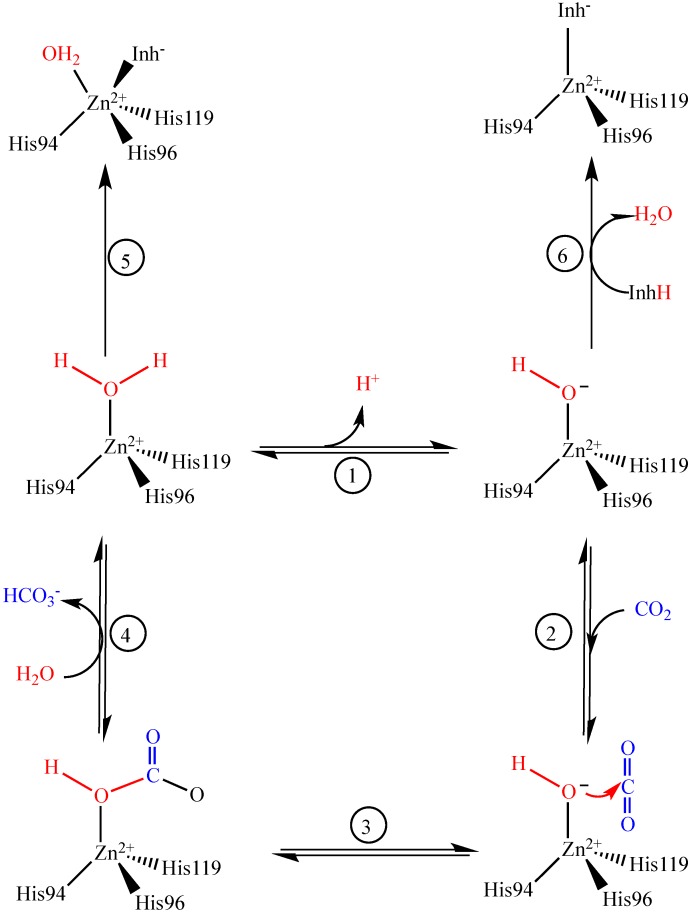
Schematic presentation of the catalytic inhibition mechanism for the CA catalyzed CO_2_ hydration.

An enzyme inhibitor is a molecule that binds to an enzyme and decreases its activity. An inhibitor can prevent a substrate from entering the active site of the enzyme or hindering catalysis. It was well known that carbonic anhydrase inhibitors (CAIs) bind to a catalytic Zn^2+^ ion in the active site of CA isoenzymes and block their activity [10,11,12,13,14,15]. CAIs are clinically used to treat glaucoma, and as anticonvulsant agents [15], diuretics [6] and antiobesity drugs [16]. Additionally, they have recently been used in the management of hypoxic tumors [17]. The first aromatic and heterocyclic sulfonamides were clinically used derivatives of acetazolamide [18]. As seen in Scheme 1, to regenerate the basic form of the enzyme, a proton is transferred from the active site to the solvent. This proton transfer may be assisted by active site residues or by buffers present in the medium. The fourth position is occupied by H_2_O at acidic pH, and is catalytically inactive. At higher pH, the water molecule binds to Zn^2+^ within the CA active site, and the proton transfer reaction transfers a proton to the solvent, leaving an -OH [11,14].

Capsaicin (*trans*-8-methyl-*N*-vanillyl-6-nonenamide) comes from the plants of genus *Capsicum* and is a pungent constituent of capsicum fruits, which are used extensively in condiments in Asian, African, and Latin American countries [19]. It is also used in a number of over-the-counter weight loss products because of its potential role in increasing metabolic activity [20]. It is the active and pungent ingredient in a wide variety of red peppers [21]. Because of its characteristic smell and taste, capsaicin is one of the most heavily consumed additives throughout the world [22].

Capsaicin is a naturally occurring alkaloid obtained from red peppers. It is generally isolated from *Capsicum annuum* and is responsible for the spiciness hotness of chili. Capsaicin and related compounds are referred to as capsaicinoids and are produced as secondary metabolites by red peppers [23]. The total intake of capsaicinoid compounds in Asian countries is estimated to be 25–200 mg/day per person given that as capsaicin accounts for 80% of the content of capsicum fruits [24]. In this study, we identify the potential inhibition profile and mechanism for human CA isoenzymes I, and II (hCA I, and II), which are widely used in the food and pharmaceutical industries.

## 2. Results and Discussion

Capsaicin is a monophenolic compound with an amide and lipophilic carbon chain on one end and a hydrophilic ring on the other. It is an amide derivative of vanillylamine and *trans*-8-methylnon-6-enoic acid. The vanillylamine moiety of capsaicin is biologically synthesized from phenylalanine. The fatty acid moiety at the other end is derived from valine [21]. Capsaicin is employed as an agricultural repellent and as an additive or colorant in the cosmetic, food and pharmaceutical industries. It has multiple pharmacological activities, including anti-inflammatory, anticancer [21], genotoxic and chemopreventive [25], antifungal and analgesic [26], neuroprotective [27], antiobesity [28], anti-apoptotic [29], and anti-epileptic effects and antioxidant activities [30]. Capsaicin is also useful in combating liver and duodenal cancers [31]. Previous research studies have shown that capsaicin induces apoptosis and cell-cycle arrest and inhibits cell proliferation in a variety of cancer cells [32]. Furthermore, it stimulates apoptotic cell death in rat trigeminal primary neurons when administered during the neonatal period [33].

**Figure 1:**
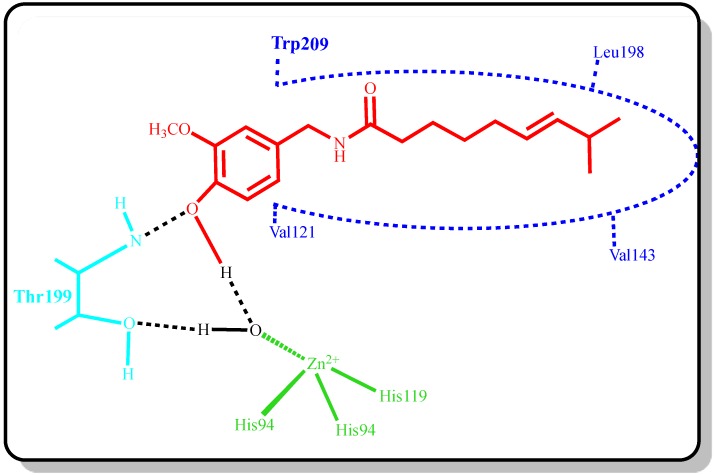
The proposed binding model of capsaicin to CA by floating to the Zn^2+^ coordinated water/hydroxide ion (-OH).

It works to speed up the body’s metabolism by activation of the sympathetic nervous system [34]. It has been shown that, in comparison to a control capsaicin supplementation control during a negative energy balance counteracts the normal decrease in energy expenditure. Moreover, the consumption of capsaicin promotes fat oxidation in negative energy balance and does not significantly increase blood pressure [35]. The chemical structure of capsaicin is shown in Figure 1, as well as an estimated binding model of capsaicin to the active site of CA. A second hydrogen bond has been modeled between the oxygen atom of phenol moiety of capsaicin and the amide NH of Thr199, an amino acid residue that is universally conserved in CAs. Thus, by binding in a non-classical way to CAs, phenols and their derivatives provide interesting leads for identifying novel types of CAIs. Capsaicin has three classical structural characteristics in one molecule, an amide with a lipophilic carbon chain on one end and a hydrophilic ring on the other. There are a large number of capsaicin analogs that contain the same basic functional groups but possess variations in one or more of the three structural characteristics [36].

CAs are involved in crucial physiological processes connected with CO_2_/HCO_3_^−^ transport and homeostasis, electrolyte secretion in a variety of tissues and organs, biosynthetic reactions including gluconeogenesis, ureagenesis and lipogenesis, respiration, calcification, tumorigenicity, and bone resorption [37]. Phenolic compounds are a class of chemicals containing of an -OH bonded directly to an aromatic hydrocarbon group and are categorized either as simple phenols or as polyphenols depending on the number of phenol units in the molecule [38,39]. Phenols, phenolic acid and phenolic derivatives were recently investigated in detail as inhibitors of the Zn^2+^-containing CA [40,41,42,43]. All CA isoforms are inhibited by three different mechanisms: (i) by coordination of the inhibitor to the Zn^2+^ from the active site of CA and replacing the Zn^2+^-bound H_2_O/-OH, which leads to a tetrahedral geometry for Zn^2+^. This geometry can also arise by the addition an inhibitor to the metal coordination sphere when the Zn^2+^ has in trigonal bipyramidal geometry [1]; (ii) by floating of the inhibitor to the Zn^2+^-bound solvent molecule, *i.e*., an H_2_O/-OH. Phenolic compounds and polyamine molecules can bind CA in this way, as shown schematically for phenol; or (iii) by inhibitor occlusion of the entrance to the active site or activator-binding site of CA [44,45,46,47].

It has been reported that simple phenol acts as an inhibitor of the Zn^2+^ containing CA isoenzymes [6,8,43,44]. Phenol binds to CA in a diverse manner when compared to the classic sulfonamide inhibitors. Sulfonamides coordinate to the Zn^2+^ ion in the CA active site by replacing the fourth non-protein ligand, which is typically a H_2_O molecule or -OH ion. By binding in a non-classical way to CAs, phenols and their derivatives constitute interesting leads for identifying novel types of CAIs [37,45,46,47]. In the present study, we report the inhibition profiles of capsaicin against the slower cytosolic isoform hCA I, and the more rapid isozymes hCA II. Capsaicin showed effective inhibition both isoforms. When examining the results, the following structure activity relationship could be easily observed.

**Figure 2:**
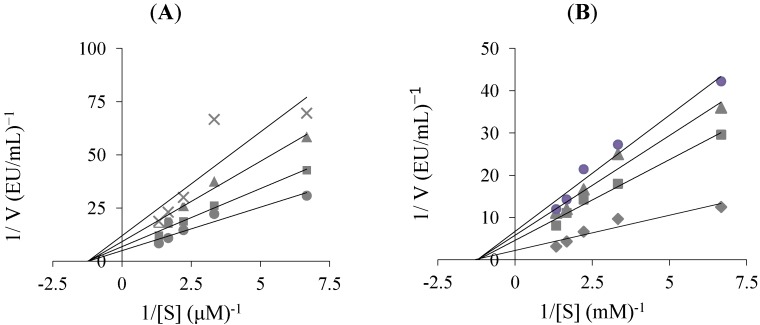
Determination of Ki values of capsaicin for human erythrocyte carbonic anhydrase I (**A**), and II (**B**) isoenzymes (hCA I, and II) by Lineweaver-Burk plots.

**Figure 3:**
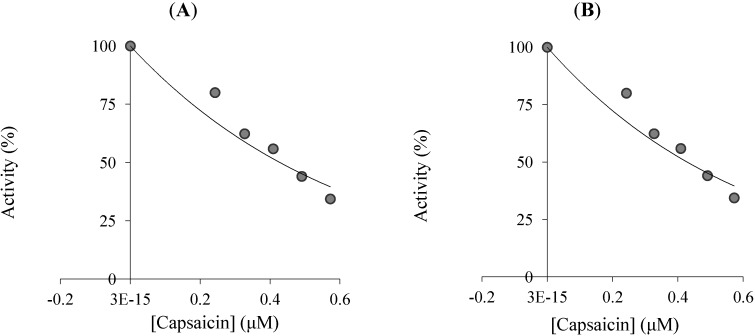
The effects of different concentrations of capsaicin on human erythrocyte carbonic anhydrase I (**A**), and II (**B**) isoenzymes (hCA I, and II).

To describe inhibitory effects, researchers often list an IC_50_ value; however, a more suitable measure is the K_i_ constant. K_i_ values were calculated from Lineweaver-Burk graphs (Figure 2), and both the Ki and IC_50_ parameters of the capsaicin were determined in this study. As shown in Table 1, Figure 2 and Figure 3, the K_i_ values for capsaicin were found, and the corresponding IC_50_ values were calculated for each CA isoenzyme. For the cytosolic isoenzyme hCA I capsaicin had an IC_50_ values of 428.04 µM and K_i_ values of 696.15 ± 59.37 µM (Table 1). For the physiologically predominant CA II, capsaicin had IC_50_ values of 316.01 µM and K_i_ values of 208.37 ± 14.38 µM. Many studies have shown that the inhibition of CA II is brought about by an inhibitor’s ability to bind to catalytic Zn^2+^ in the CA’s active site and mimic to tetrahedral transition state [6,11,14]. Thus, in Figure 1, we illustrate a binding model between capsaicin and the enzyme’s active site. There are important differences in inhibition between the two isoenzymes. The main difference in the active site architectures of two isozymes is due to the presence of more histidine residues in the CA I isoform [14]. In addition to the Zn^2+^ ligands (His 94, His 96, and His 119), discussed in the introduction, His 64 of CA I plays an important role in catalysis.

**Table 1:** The inhibition profile of capsaicin on purified hCA I, and hCA II from human erythrocytes by Sepharose-4B-l-tyrosine-sulfanilamide affinity chromatography.

Kinetic Parameters	hCA I	hCA II
IC50 (µM)	428.04	316.01
Ki (µM)	696.15 ± 59.37	208.37 ± 14.38
Inhibition type	Uncompetitive	Uncompetitive

Another important difference between the two isozymes is that CA II contains a histidine cluster, consisting of the following residues: His 64, His 4 His 3, His 10, His 15, and His 17 which is absent in CA I. Hence, these two isozymes exhibit different affinities for the inhibitors. In general, CA II has a higher affinity for the inhibitor than CA I [14].

hCA I is highly abundant in red blood cells and found in many tissues although its precise physiological function is unknown. CA I is associated with cerebral and retinal edema, and the inhibition of CA I inhibition may be a valuable tool for fighting these conditions. The physiologically predominant cytosolic isoform hCA II is ubiquitous and associated with several diseases including epilepsy, edema, glaucoma, and altitude sickness [17,48].

Acetazolamide is a well-known example of a clinically established carbonic anhydrase inhibitor [49,50] and in recent years we have reported on the strong inhibition of both human cytosolic CA I, and II. CA inhibitory effects are also exhibited by a wide spectrum of phenolic compounds including melatonin [9], morphine [51], vitamin E [52], CAPE [50], antioxidant phenols [47], phenolic acids [41], natural product polyphenols and phenolic acids [43], natural phenolic compounds [10], antioxidant polyphenol products [45,46], (3,4-dihydroxyphenyl)(2,3,4-trihydroxyphenyl)methanone and its derivatives [13], natural and synthetic bromophenols [53], novel sulfonamide derivatives of aminoindanes and aminotetralins [7], novel phenolic sulfamides [54], novel phenolic benzylamine derivatives [5], novel sulfamide analogues of dopamine related compounds [3], new benzotropone derivatives [11], brominated diphenylmethanone and its derivatives [55] and novel sulfamides and sulfonamides incorporating tetralin scaffold [4] have been reported. These extensive studies indicate the importance of CA I, and II isoenzyme inhibitors.

## 3. Experimental

*Biochemistry*

Both of the CA isoenzymes were purified by Sepharose-4B-L tyrosine-sulphanilamide affinity chromatography [51] accordance to previous studies [52,56]. For purification, the lysate was adjusted to pH 8.7 with Tris. Then, an aliquot of the lysate was applied to the affinity column and proteins content in the eluates was observed spectrophotometrically at 280 nm. Sodium dodecyl sulphate-polyacrylamide gel electrophoresis (SDS-PAGE) was performed after purification of the enzymes. The isoenzymes purities were determined by SDS-PAGE and a single band was observed for each CA isoenzyme [57]. This method has been described previously [58] and was performed using acrylamide in the running (10%) and the stacking gel (3%) with SDS (0.1%) [59,60].

Both CA isoenzyme activities were determined according to Verpoorte *et al*. [61] as described previously [62,63]. The absorbance change at 348 nm was observed over a period of 3 min at room temperature (25 °C) using a spectrophotometer (UVmini-1240 UV-VIS spectrophotometer, Shimadzu, Kyoto, Japan), before and after adding the sample. One unit of enzyme activity was expressed as 1 mol/L of released *p*-nitrophenol per minute at 25 °C [64]. The quantity of protein was spectrophotometrically determined at 595 nm during purification the Bradford method [65] and bovine serum albumin (BSA) was used as the standard protein [66,67,68].

The inhibition effect of capsaicin on CA isoenzymes was measured the hydrolysis of *p*-nitrophenyl acetate (PNA) by CA to *p*-nitrophenol; *p*-nitrophenol can be quantified spectrophotometrically [12]. The CA-catalysed reaction of CO_2_ hydration was first observed in the absence of capsaicin; the rates were measured and used as a control for the CA isoenzymes. Then the same reaction was measured in the presence of capsaicin. The percent inhibition was determined with (%) = [100 − (ATC/AC) × 100]; A_TC_ is the absorbance of the sample containing capsaicin and A_C_ is the absorbance of the control sample. Activity (%)-[capsaicin] graphs were drawn and the half maximal inhibitory concentration (IC_50_) values of capsaicin exhibiting more than 50% inhibition of CA were calculated after suitable dilutions. IC_50_ is a measure of the potency of capsaicin in inhibiting CA isoenzyme activity. In addition to these values, the K_i_ values for capsaicin were determined for each isoenzyme. To determine the K_i_ values, capsaicin was tested at three different concentrations. K_i_ is the binding affinity constant of the inhibitor. In these experiments, NPA was used as the substrate at five different concentrations and Lineweaver-Burk curves were drawn [69] in detail as described previously [70,71,72,73].

## 4. Conclusions

Capsaicin exhibited unique inhibition profiles against both CA isoform I, and II. These results indicate that, despite the high homology between these two CAs, they do not display similar activity. The logic of working with capsaicin was first to identify a potent CA inhibitor because phenolic compounds with aromatic rings have been previously identified as inhibitors of CA. In this study, micromolar levels of K_i_ and IC_50_ values in the micromolar range were observed for capsaicin. We show that capsaicin is a selective inhibitor of both cytosolic CA isoenzymes. These results clearly indicate the potential use for bioactive phenolic capsaicin in identifying more CA inhibitors and for eventually targeting additional isoforms.
